# Supplementary material for: Antimicrobial Activity of Essential Oils Evaluated In Vitro against Escherichia coli and Staphylococcus aureus
Source: Antibiotics (Basel). 2022 Jul 20;11(7):979. doi: 10.3390/antibiotics11070979 (PMC9311876; doi:10.3390/antibiotics11070979)
Supplement: Supplementary file 1 [file antibiotics-11-00979-s001.zip › antibiotics-1806348-supplementary.pdf]

Supplementary files

Table S1. Chemical composition of tested *Essential Oil*

[a]: standard compounds. Linear retention index (LRI) on HP-5MS column was experimentally determined using a homologous series of C10-C40 alkanes standard mixture (Van den Dool and Kratz, 1993). Arithmetic index (AI) was taken from Adams (2007) and/or the NIST 2017 Database (NIST 17, 2017. Mass Spectral Library (NIST/EPA/NIH). Gaithersburg, USA: National Institute of Standards and Technology. Last access 12_2021). Similarity index/mass spectrum (SI/MS) was compared with data reported on NIST 2017 Database and were determined as reported by Koo et al. (2013), and Wan et al. (2002). Relative percentage values are means of three determinations with a structural equation modeling (SEM) in all cases below 10%. a) *Citrus lemon* Essential Oil; b) *Pinus sylvestris* Essential Oil; c) *Foeniculum vulgare* Essential Oil; d) *Ocimum basilicum* Essential Oil; e) *Melissa officinalis* Essential Oil; f) *Thymus vulgaris* Essential Oil; g) *Zingiber officinalis Rosc*. Essential Oil

a)

N	Components	LRI	AI	*Citrus lemon*	
				**Area±SEM**	**SI/MS**
1	Ethyl propanoate	714	714	0.10±0.010	91
2	α-pinene [a]	930	931	2.42±0.52	95
3	β-thujene	968	968	1.94±0.20	86
4	β-pinene[a]	982	980	14.55±1.05	94
5	limonene[a]	1030	1032	53.19±5.11	93
6	γ-terpinene[a]	1062	1064	5.91± 1.02	94
7	terpinolene	1083	1085	0.22±0.020	96
8	β-linalool[a]	1100	1101	0.21±0.020	91
9	(E)-p-menth-2,8-dien-1-ol	1122	1123	0.10±0.0020	80
10	limonene oxide, cis-	1130	1131	1.01±0.87	96
11	limonene oxide, trans-	1138	1138	0.68±0.080	91
12	α-terpineol	1178	1179	0.30±0.020	80
13	cis-carveol	1222	1222	0.26±0.020	96
14	citral[a]	1240	1240	3.76±0.90	96
15	Δ−carvone	1242	1242	0.12±0.010	93
16	nerol acetate	1363	1364	0.83±0.054	91
17	geranyl aceate	1384	1385	0.87±0.056	91
18	caryophyllene[a]	1415	1415	0.13±0.012	99
19	α-bergamotene	1431	1430	0.21±0.020	87
20	β-bisabolene [a]	1504	1506	0.32±0.040	95
21	caryophylleneoxyde	1596	1592	0.31±0.050	91
	% Characterized	/	/	87.44	/
	Others	/	/	12.56	/

b)

N	Components	LRI	AI	*Pinus sylvestris*	
				**Area±SEM**	**SI/MS**
1	Ethyl propanoate	714	714	0.11±0.010	91
2	santene	892	890	0.17±0.020	90
3	cyclene	920	919	0.19±0.030	91
4	α-pinene [a]	930	931	29.05±3.03	94
5	camphene [a]	952	952	1.64±0.30	97
6	β-thujene	968	968	1.24±0.23	78
7	β-pinene[a]	982	980	17.16±1.25	94
8	α-terpinene	1014	1014	0.70±0.050	91
9	3-carene[a]	1015	1016	13.12±2.50	94
10	o-cymene	1021	1021	1.10±0.81	95
11	limonene[a]	1030	1032	9.75±1.20	99
12	terpinolene	1083	1085	2.09±0.87	96
13	α-terpineol	1178	1179	0.52±0.030	86
14	p-cymen-8-ol	1183	1183	0.37±0.030	90
15	myrtenol	1196	1196	0.17±0.010	91
16	eucarvone	1245	1245	0.12±0.010	91
17	bornylacetate[a]	1288	1289	5.66±1.25	99
18	α-cubebene	1347	1348	0.13±0.010	97
19	longifolene	1412	1412	1.38±0.49	99
20	caryophyllene[a]	1415	1415	4.93±0.88	99
21	humulene	1452	1451	0.47±0.025	97
22	caryophyllene oxyde	1596	1592	1.68±0.29	91
	% Characterized	/	/	91.75	/
	Others	/	/	8.25	/

c)

N	Components	LRI	AI	*Foeniculum vulgare*	
				**Area±SEM**	**SI/MS**
1	Ethyl propanoate	714	714	0.11±0.010	91
2	α-pinene [a]	930	931	6.38±0.89	97
3	camphene [a]	952	952	0.12±0.010	94
4	β-pinene[a]	982	980	0.65±0.070	95
5	β-myrcene	990	991	1.10±0.080	91
6	α-phellandrene[a]	1002	1003	5.13±0.55	91
7	o-cymene	1021	1021	1.00±0.070	95
8	trans-β-ocimene	1028	1027	0.58±0.050	97
9	limonene[a]	1030	1032	5.12±1.01	94
10	camphene hydrate	1147	1147	1.98±0.85	94
11	γ-terpinene[a]	1062	1064	0.15±0.020	96
12	estragole[a]	1198	1198	1.53±0.074	98
13	p-anisaldehyde	1271	1270	0.61±0.030	94
14	anethole[a]	1283	1284	58.68±3.87	98
15	sabinyl acetate	1291	1291	0.12±0.010	64
16	α-bergamotene	1431	1430	0.10±0.012	93
	% Characterized	/	/	83.36	/
	Others	/	/	16.64	/

d)

N	Components	LRI	AI	*Ocimum basilicum*	
				**Area±SEM**	**SI/MS**
1	eucalyptol	1023	1023	0.29±0.020	99
2	cis-linalol oxide	1066	1066	0.44±0.03	90
3	β-linalool[a]	1100	1101	16.98±2.55	97
4	menthol	1169	1169	0.57±0.040	91
5	estragole[a]	1198	1198	73.04±6.09	98
6	citral[a]	1240	1240	1.10±0.080	96
7	caryophyllene[a]	1415	1415	0.43±0.020	99
8	α-bergamotene	1431	1430	3.18±0.40	94
9	humulene	1452	1451	0.23±0.023	94
10	(E)-β-famesene	1459	1459	0.41±0.020	97
11	β-bisabolene [a]	1504	1506	0.13±0.0010	93
12	trans-4-methoxy cinnamaldehyde	1568	1570	0.92±0.080	97
13	caryophyllene oxyde	1596	1592	0.22±0.012	80
	% Characterized	/	/	97.94	/
	Others	/	/	2.06	/

e)

N	Components	LRI	AI	*Melissa officinalis*	
				**Area±SEM**	**SI/MS**
1	α-pinene [a]	930	931	0.34±0.040	96
2	camphene [a]	952	952	0.52±0.050	96
3	β-thujene	968	968	0.20±0.010	94
4	β-pinene[a]	982	980	0.65±0.050	91
5	eucalyptol [a]	1023	1023	1.23±0.52	98
6	trans-β-ocimene	1028	1027	0.11±0.050	97
7	limonene[a]	1030	1032	4.32±0.99	94
8	4-nonanone (propyl amyl ketone)	1052	1053	0.27±0.012	91
9	β-linalool[a]	1100	1101	0.96±0.25	97
10	citronellal	1168	1170	0.55±0.050	93
11	α-terpineol	1178	1179	0.27±0.015	80
12	citronellol	1220	1221	0.34±0.060	95
13	citral[a]	1240	1240	42.7±3.45	96
14	geraniol	1253	1254	2.13±1.03	97
15	α-cubebene	1347	1348	0.40±0.020	98
16	eugenol	1358	1359	0.15±0.010	93
17	β-ylangene	1367	1368	0.13±0.012	93
18	α-copaene	1374	1375	0.86±0.090	99
19	geranyl aceate	1384	1385	1.95±0.10	91
20	β-elemene (-)	1394	1394	0.16±0.012	83
21	caryophyllene[a]	1415	1415	25.29±1.05	99
22	trans-isoeugenol	1426	1427	0.27±0.012	95
23	α-bergamotene	1431	1430	0.14±0.010	87
24	humulene	1452	1451	4.39±0.92	97
25	alloaromadendrene	1458	1458	0.16±0.010	90
26	α-amorphene	1483	1484	0.84±0.080	97
27	α-farnesene [a]	1509	1508	0.13±0.011	93
28	caryophyllene oxyde	1596	1592	2.18±0.87	91
	% Characterized	/	/	91.64	/
	Others	/	/	8.36	/

f)

N	Components	LRI	AI	*Thymus vulgaris*	
				Area±SEM	SI/MS
1	Ethyl propanoate	714	714	0.10±0.010	86
3	cyclene	920	919	0.13±0.090	94
4	α-pinene [a]	930	931	1.81±0.10	95
5	camphene [a]	952	952	1.89±0.11	96
6	β-thujene	968	968	0.71±0.060	93
7	mushroom alcohol	975	975	0.37±0.020	83
8	β-pinene[a]	982	980	0.56±0.030	94
9	β-myrcene	990	991	1.42±0.080	86
10	α-phellandrene[a]	1002	1003	0.15±0.010	91
11	o-cymene	1021	1021	19.64±1.50	95
12	eucalyptol [a]	1023	1023	0.89±0.050	99
13	limonene[a]	1030	1032	0.60±0.040	91
14	γ-terpinene[a]	1062	1064	8.83±1.03	94
15	terpinolene	1083	1085	1.33±0.88	97
16	β-linalool[a]	1100	1101	4.07±1.02	97
17	camphor	1145	1146	1.69±0.85	98
18	terpineol, cis-β-	1147	1147	0.13±0.010	90
19	endo-borneol	1166	1167	1.85±0.70	97
20	terpinen-4-ol	1171	1171	1.83±0.83	96
21	α-terpineol	1178	1179	0.12±0.010	86
22	anisole	1234	1235	0.39±0.060	90
23	anisole, 2-isopropyl-4-methyl-	1244	1244	0.42±0.050	94
24	thymol[a]	1290	1290	47.01±3.32	94
25	caryophyllene[a]	1415	1415	2.18±0.87	99
26	caryophyllene oxyde	1596	1592	0.58±0.030	91
	% Characterized	/	/	98.70	/
	Others	/	/	1.30	/

g)

N	Components	LRI	AI	*Zingiber officinalis Rosc.*	
				**Area±SEM**	**SI/MS**
1	hexanal	805	801	0.15 ± 0.013	91
2	α-pinene [a]	930	931	1.73 ± 0.12	97
3	camphene [a]	952	952	4.74 ± 0.27	96
4	β-pinene	976	974	0.71 ± 0.11	97
5	prenylacetone	987	986	0.41 ± 0.074	90
6	α-phellandrene [a]	1003	1002	4.35 ± 0.57	87
7	eucalyptol [a]	1023	1023	1.93 ± 0.42	98
8	terpinolene	1087	1086	0.17 ± 0.023	96
9	linalol	1094	1095	0.23 ± 0.091	91
10	endo borneol [a]	1166	1167	1.01 ± 0.12	97
11	α-terpineol	1187	1186	0.52 ± 0.24	91
12	decanal	1206	1201	0.13 ± 0.065	87
13	citronellol	1228	1223	0.13 ± 0.061	96
14	geraniol	1252	1249	0.25 ± 0.098	90
15	bornyl acetate	1284	1287	0.13 ± 0.096	98
16	2-undecanone	1294	1293	0.22 ± 0.10	94
17	α-cubebene	1348	1345	0.72 ± 0.13	95
18	cyclosativene	1364	1362	0.58 ± 0.21	94
19	2-butyl-2-octenal	1375	1372	0.17 ± 0.096	96
20	geranyl acetate	1385	1379	0.49 ± 0.17	91
21	caryophyllene	1425	1417	0.14 ± 0.098	93
22	α-bergamotene	1430	1432	0.17 ± 0.065	93
23	γ-elemene	1439	1434	0.56 ± 0.19	98
24	β-farnesene	1451	1454	0.73 ± 0.13	97
25	alloaromadendrene	1457	1458	0.56 ± 0.15	92
26	α-elemene	1467	1469	0.21 ± 0.096	90
27	α-curcumene [a]	1479	1481	15.2 ± 1.12	99
28	zingiberene [a]	1495	1493	32.1 ± 1.78	93
29	α-muurolene	1502	1500	0.29 ± 0.11	97
30	α-farnesene [a]	1506	1505	7.23 ± 0.98	93
31	β-bisabolene [a]	1507	1505	2.29 ± 0.25	93
32	β-sesquiphellandrene [a]	1523	1521	10.91 ± 1.10	90
33	cubenene	1529	1533	1.27 ± 0.27	93
34	cis-sesquisabinene hydrate	1539	1542	0.41 ± 0.23	86
35	β-elemol	1542	1548	0.51 ± 0.12	91
36	β-nerolidol	1562	1561	0.87 ± 0.23	90
37	trans-sesquisabinene hydrate	1579	1577	0.39 ± 0.29	93
38	beta-eudesmol	1653	1649	0.25 ± 0.11	99
	% Characterized	/	/	92.85	/
	Others	/	/	7.15	/
